# First Descriptions of Larva and Pupa of *Bagous claudicans* Boheman, 1845 (Curculionidae, Bagoinae) and Systematic Position of the Species Based on Molecular and Morphological Data

**DOI:** 10.3390/insects10060166

**Published:** 2019-06-10

**Authors:** Rafał Gosik, Miłosz A. Mazur, Natalia Sawka-Gądek

**Affiliations:** 1 Department of Zoology, Maria Curie–Skłodowska University, Akademicka 19, 20–033 Lublin, Poland; r.gosik@poczta.umcs.lublin.pl; 2 Institute of Biology, University of Opole, Oleska 22; 45–052 Opole, Poland; 3 Institute of Systematics and Evolution of Animals Polish Academy of Sciences; Sławkowska 17, 31–016 Kraków, Poland; sawka@isez.pan.krakow.pl

**Keywords:** Weevils, Coleoptera, Curculionidae, Bagoini, *Bagous*, taxonomy, morphology, larva, pupa, biology, COI, DNA barcoding

## Abstract

In this paper, the mature larva and pupa of *Bagous claudicans* are described and illustrated for the first time. Measurements of younger larval instars are also given. The biology of the species is discussed in association with larval morphology and feeding habits. Overall larval and pupal morphological characters of the genus *Bagous* are presented. Confirmation of the larva identification as *Bagous claudicans* species was conducted by cytochrome oxidase I (*COI*) sequencing. DNA barcoding was useful for specimen identification of larval stages. The systematic position of the species within the *Bagous collignensis*-group, based on morphological and molecular results, is also discussed.

## 1. Introduction

The globally distributed (except for Central and South America) weevil genus *Bagous* Germar, 1817 includes about 300 valid species, of which approximately 130 occur in the Palaearctic region, 82 in the Western Palaearctic, and 31 in Central Europe [1,2,3,4,5,6,7]. This makes it one of the most numerous among the weevil genera and the largest group of hydrophilous beetles, which comprise less than 1% of all known coleopteran species [8]. The genus has received much taxonomic revision (e.g., [4,9,10,11,12]), and a comprehensive classification of the tribe Bagoini Thomson, 1859 was recently proposed following a global phylogenetic analysis performed by Caldara et al. [13].

The adult *Bagous* weevils present a rather uniform group characterized by: (1) a small- to medium-sized body (1.2–8.9 mm) densely covered with granulate and pitted scales often with waterproof coating; (2) rostrum shorter than pronotum with distinct, well-developed dorsolateral sulcus above scrobes; (3) antennae inserted near apex of rostrum; (4) tibiae slender, ventrally sinuate or bisinuate with a conspicuous uncus; (5) third tarsomere linear, subcordate or (most commonly) indistinctly bilobed; (6) dorsal surface of penis body fully sclerotised, at least basally [4,8,10,12,14]. 

Only several of the Bagoini can be regarded as well-known with reference to their biology and plant association (e.g., *B. nodulosus* Gyllenhal, 1836, *B. glabrirostris* (Herbst, 1795)). Based on records collected to date, most are regarded as nocturnal, inhabiting aquatic biotopes, e.g., ponds, old riverbeds, swamps, floodplain meadows and forests, where many of them develop on submerged vegetation. But several species (such as *B. tempestivus* (Herbst, 1795), *B. lutulosus* (Gyllenhal, 1827) or *B. diglyptus* Boheman, 1845) also settle in more or less dry, terrestrial habitats or even xerothermic communities (e.g., *B. aliciae* Cmoluch, 1983) [4,6,15]. The majority of Bagoini are known as monophagous or very narrowly oligophagous beetles preferring Dicotyledones, exceptions include *B. glabrirostris* (Herbst, 1795) and *B. limosus* Gyllenhal, 1827, which feed on many plant species recruited from different families, and *B. lutulentus* (Gyllenhal, 1813) that live on horsetails, *Equisetum fluviatile* L. (Equisetales) [4,6,15,16,17,18,19]. 

As stenotopic species associated with the clear water-dependent natural biotopes (threatened nowadays on devastation), a great number of *Bagous* species are endangered or close to becoming extinct (such as *B. petro* (Herbst, 1785), *B. elegans* (Fabricius, 1801), and *B. nupharis* Apfelbeck, 1906 (= *B. rotundicollis* Boheman, 1845) [20,21,22,23]. Thus, some of them are used as habitat change indicators [22]. In addition to degradation of natural habitats, the low dispersal capability of weevils is an important factor in the vanishing of their populations, the species are also sometimes representing a relic of the Ice Age or an endemic element [12,13,21,22,24]. On the other hand, several species have been used as biological control agents against noxious or invasive plants (e.g., *B. affinis* Hustache, 1926 against *Hydrilla verticillata* (L. f.) Royle, *B. nodulosus* against *Butomus umbellatus* L. or *B. longitarsis* Thomson, 1868 against *Myriophyllum spicatum* L.) [25,26,27,28,29,30]. 

Within the Bagoini species, the morphology of larval instars has been described in 15 species: *Hydronomus alismatis* Schoenherr 1825 [31], *B. australasiae* Blackburn 1894 [32], *B. binodulus* (Gyllenhal, 1813) [18], *B. brevis* Gyllenhal 1836 [5], *B. collignensis* (Herbst, 1797) [18], *B. frit* (Herbst, 1795) [33], *B. frivaldszkyi* Tournier, 1874, *B. lutulentus* [18], *B. nodulosus* [34], *B. robustus* Brisout de Barneville, 1863 [35], *B. rufimanus* Pericart, 1989 [36], *B. subcarinatus* Gyllenhal, 1836 [37], *Bagous elegans*, *B. aliciae* and *B. lutulosus* [38,39]; while the pupae have been described in eight species [5,18,33,34,35,36,37,38,39]. These sparse records are enough to establish that the *Bagous* genus is highly diverse in both the morphology of immature and the mode of larval feeding. For example, larvae of *B. frivaldszkyi* and *B. nodulosus* live inside submerged parts of plant tissue while larvae of *B. lutulentus* in emerged portions. Moreover, the larvae of *B. binodulus*, *B. brevis*, *B. lutulosus*, *B. aliciae* are exophagous, while larvae of *B. alismatis* are leaf-miners. Pupation takes place in the larval chamber, seldom in the soil. The overwintering stage is always the imago [5,17,18,19,22,23,31,35]. 

## 2. Materials and Methods

### 2.1. Materials

All specimens used in this study (7 exx of adults, 3 exx of first instars larva, 9 exx of mature larva, and one pupa (♀)) were collected from one place:

Poland: Katowice, downtown plantings of *Sedum maximum* (L.) Suter, 50°15′48.2″ N 19°02′02.5″ E.

Specimens have been found inside plant tissue: mainly root collar, underground parts of roots and stems.

One larva and seven adult specimens were used for DNA extraction and molecular studies. Most of the larval forms and one pupa were used to prepare morphological description of the immature stages. The remainder of the specimens (morphological vouchers) were deposited in the collections of the Department of Zoology, Maria Curie–Skłodowska University (Lublin, Poland). The remainder of the specimens after DNA extraction (two undamaged specimens) were deposited in the Voivodeship Plant Health and Seed Inspection Service in Katowice (Poland). Molecular vouchers are deposited in the Institute of Systematics and Evolution of Animals Polish Academy of Sciences (Krakow, Poland).

### 2.2. Methods

#### 2.2.1. Morphological Studies

All specimens described were fixed in 75% ethanol and examined under an optical stereomicroscope (Olympus SZ 60 and SZ11, Olympus, Tokyo, Japan) with calibrated ocular graticules. Measurements of larval instars were made for body length (BL), body width (BW) (at abdominal segment 2), and width of the head capsule (HW). In pupae, body length (BL), body width (BW) (at the level of middle legs), and width of pronotum (THW) were given.

The observations of chaetotaxy and measurements were conducted using a light compound microscope with calibrated ocular graticules. Drawings and outlines were made using a drawing tube (MNR–1) installed on a stereomicroscope (Biolar, Polskie Zakłady Optyczne, Warsaw, Poland) and processed by computer software (Corel Photo-Paint X7, Corel Draw X7). Photos were taken using an Olympus BX63 microscope and processed by Olympus cellSens Dimension software. The larvae selected for pictures using SEM (scanning electron microscope) were at first dried in absolute ethyl alcohol (99.8%), rinsed in acetone, critical-point dried and then gold-plated. TESCAN Vega 3 SEM was used for the examination of selected structures. General terminology and chaetotaxy follow Anderson [40], May [32], Marvaldi [41,42,43,44], and Skuhrovec et al. [45], with antennae terminology following Zaharuk [46]. 

#### 2.2.2. DNA Extraction, Amplification and Sequencing 

Eight specimens (one larva and seven imagines) were used for molecular analysis. Before DNA extraction, all specimens were cleaned using ethanol and distilled water in order to reduce the risk of contamination. DNA was extracted from whole insect body (without any infraction in case of two specimens, they were retained as morphological vouchers, see above), as well as without protocol modification using the NucleoSpin Tissue kit (Macherey–Nagel, Düren, Germany). To elute purified DNA, 100 μL of Elution Buffer were applied onto the silica membrane. To amplify the barcode fragment of cytochrome oxidase gene, the following primer pair was used: LepF1 and LepR1 [47]. 

Polymerase chain reaction (PCR) amplification for all DNA fragments analyzed was carried out in a final volume of 20 μL containing 30 ng of DNA, 1.25 U GoTaq G2 Flexi (Promega, Madison, WI, USA), 0.8 μL of 20 μM of each primer, 4 μL of 5x PCR buffer, and 0.4 μL of 10 mM dNTPs in a Mastercycler ep system (Eppendorf, Hamburg, Germany). The cycling profile for the PCR was as follows: 95 °C for 2 min, 35 cycles of 95 °C for 1 min, 50 °C for 1 min, 72 °C for 1 min, and a final extension period of 72 °C for 7 min.

In order to assess the quality of the amplification, PCR products were electrophoresed in 1% agarose gel for 45 min at 85 V with a DNA molecular weight marker (Mass Ruler Low Range DNA Ladder, Thermo Fisher Scientific, Waltham, MA, USA). PCR products were purified using Exo–BAP Mix (EURx, Gdańsk, Poland).

Samples were sequenced in both directions using the same primers as for PCR reactions in combination with the Bright Dye Terminator Reaction Ready Mix v. 3.1 (Nimagen, Nijmegen, the Netherlands) using the chain termination reaction method [48]. The sequencing reaction was conducted using the PCR product in a total volume of 10 μL, containing 1 μL Bright Dye Terminator Reaction Ready Mix v. 3.1 (Nimagen), 1.5 μL 5× sequencing buffer (Nimagen), 3.2 mol/ μL primer solution, and 3 μL purified PCR product. The cycle-sequencing profile was 3 min at 94 °C followed by 30 cycles of 10 s at 96 °C; 5 s at 50 °C; and 2 min at 60 °C.

Sequencing products were precipitated using ExTerminator (A&A Biotechnology, Poland), and were separated on an ABI PRISM 377 DNA Sequencer (Applied Biosystems, USA). Sequences for *Bagous claudicans* are available in the GenBank database under the following accession numbers MK533683–MK533690.

#### 2.2.3. Sequence and Data Analysis 

Raw chromatograms were evaluated and corrected in Geneious R10 (https://www. geneious.com). The possibility of having sequenced numts [49,50] was rejected by translating DNA data into amino acid sequences using invertebrate genetic code within Geneious R10.

The nucleotide sequences were verified using BLAST (Basic Local Alignment Search Tool) searches of NCBI (National Center for Biotechnology Information) (http://blast.ncbi.nlm.nih.gov/Blast.cgi). The alignment of the studied sequences was performed using the MAFFT [51] plugin within Geneious R10. 

The mtCOI sequences for the remaining Bagous species were retrieved from GenBank to perform the phylogenetic analysis.

Phylogenetic trees were reconstructed using Bayesian inference (BI) and maximum likelihood (ML). The most appropriate substitution model of evolution was inferred using jModelTest [52]. The Akaike information criterion was preferred over the hierarchical likelihood ratio test to compare the various models as recommended by Posada and Buckley [53]. Bayesian analyses were performed with MrBayes 3.2 [54], consisting of two Markov chain Monte Carlo (MCMC) analyses run for 1,000,000 generations, with trees sampled every 100 generations and using four chains and default priors. Convergence of each run was also visually inspected using Tracer [55]. An initial10% of sampled trees were discarded as burn-in and a majority-rule consensus tree was obtained in TreeAnnotator v1.10.1 [56] 

ML analysis was performed using PhyML3.0 [57]. Support values were computed with 1000 bootstrap replications. All trees were visualized with TreeView v.1.6.6 (http://tree.bio.ed.ac.uk/software/figtree/) and graphically edited in CorelDraw X8 (CorelDraw Corporation, Ottawa, ON, Canada). 

Pairwise distances were calculated using MEGA v.7 [58]. Analysis of other metrics (such as haplotype and nucleotide diversities) was not performed due to the low number of available sequences per species.

## 3. Results

### 3.1. Description of the Larva of Bagous claudicans

**Figure 1:**
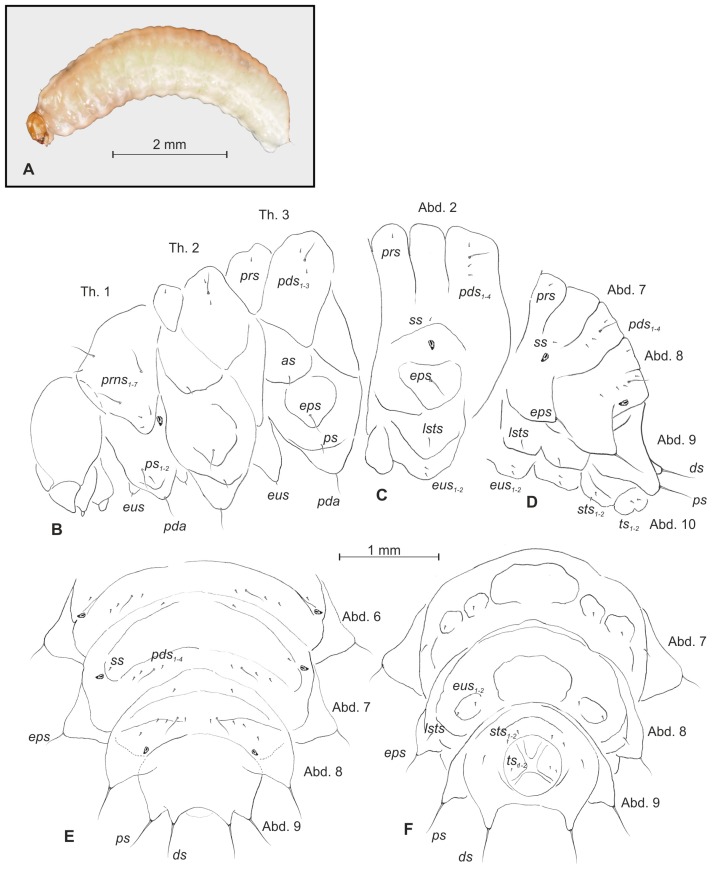
*Bagous claudicans* mature larva, habitus and chaetotaxy. (**A**)—habitus; (**B**)—lateral view of thoracic segments; (**C**)—lateral view of second abdominal segment; (**D**)—lateral view of the abdominal segments 7–10; (**E**)—dorsal view of abdominal segments 6–10; (**F**)—ventral view of abdominal segments 7–10 (Th. 1–3—thoracic segments 1–3, Abd. 1–10—abdominal segments 1–10, setae: *as*—alar, *ps*—pleural, *eps*—epipleural, *ds*—dorsal, *lsts*—laterosternal, *eus*—eusternal, *pda*—pedal, *pds*—postdorsal, *prns*—pronotal, *prs*—prodorsal, *ss*—spiracular, *sts*—sternal, *ts*—terminal).

**Figure 2:**
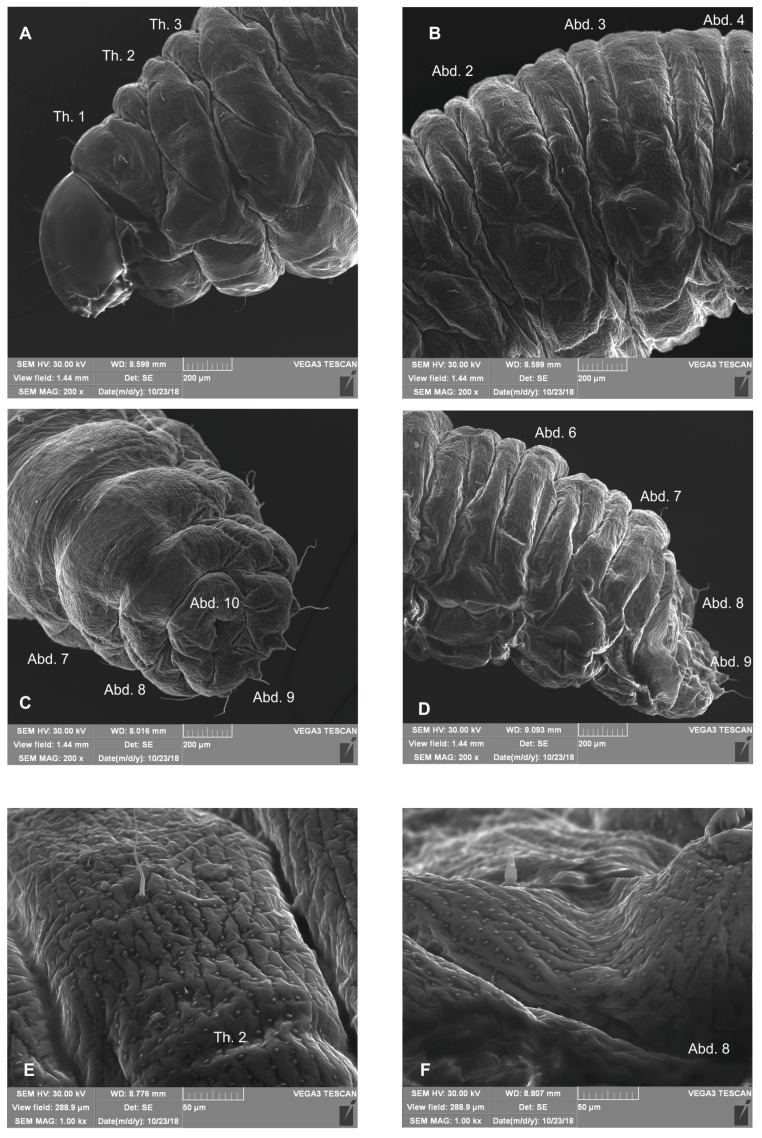
*Bagous claudicans* mature larva, habitus and cuticle, scanning electron microscope (SEM) photography. (**A**)—lateral view of thoracic segments; (**B**)—lateral view of abdominal segments; (**C**)—last abdominal segments, ventral view; (**D**)—last abdominal segments, lateral view; (**E**)—structure of cuticle on thoracic segment; (**F**)—structure of cuticle on abdominal segment 8 (Th. 1–3—thoracic segments 1–3, Abd. 1–10—abdominal segments 1–10).

*General morphology* (Figure 1A–F, Figure 2A–F). All thoracic and abdominal segments were white–yellow (Figure 1A). Cuticle densely covered with asperities (Figure 2E,F). Pronotal area of first thoracic segment feebly sclerotized, light yellow. Body very slender, elongated, slightly curved (Figure 1A), round in cross section. Prothorax slightly shorter than the subequal meso- and metathorax. Abd. 1–6 of almost equal length, slightly longer than metathorax; Abd.7–9 decreasing gradually to the terminal parts of the body; Abd. 10 reduced to four anal lobes of unequal size (dorsal the biggest, ventral the smallest, lateral lobes slightly smaller. Dorsal parts of Abd. 1–7 divided into three lobes, Abd. 8 with two dorsal lobes. Lateral folds of Abd. 1–9 well isolated, on segments 6–9 developed into conical protuberances. Nine pairs of bicameral spiracles, first pair placed on anterior margin of pronotum, next seven pairs latero-medial, the last pair placed dorso-laterally on Abd. 8 (Figure 1B–F, Figure 2A–D). 

*Chaetotaxy* (Figure 1B–F, Figure 2A–D). Setae light yellow, fine, trichiform, varying in size, feebly developed, sometimes poorly distinguished from asperities (Figure 2E, F). Prothorax on each side (Figure 1B) with seven *prns* of unequal length (three long and four short, all located on sclerotized shield), two *ps* of various length, and one short *eus*. Mesothorax (Figure 1B) on each side have one minute *prs*, three *pds* (first and third minute, second relatively long), one medium *as*, one long *eps*, one medium *ps* and one minute *eus*. Chaetotaxy of metathorax (Figure 1B) similar to mesothorax. Each pedal area of thoracic segments has one long *pda*. Abd. 1–8 with one minute *prs*, four *pds* (first, third, and fourth minute, second long), one minute *ss*, one long *eps*, one medium *lsts* and two minute *eus*. Abd. 9 on each side has one very long *ds*, one long *ps*, and two minute *sts* (Figure 1C–F). Each lateral lobe of Abd. 10 has two minute *ts* (Figure 1F). 

**Figure 3:**
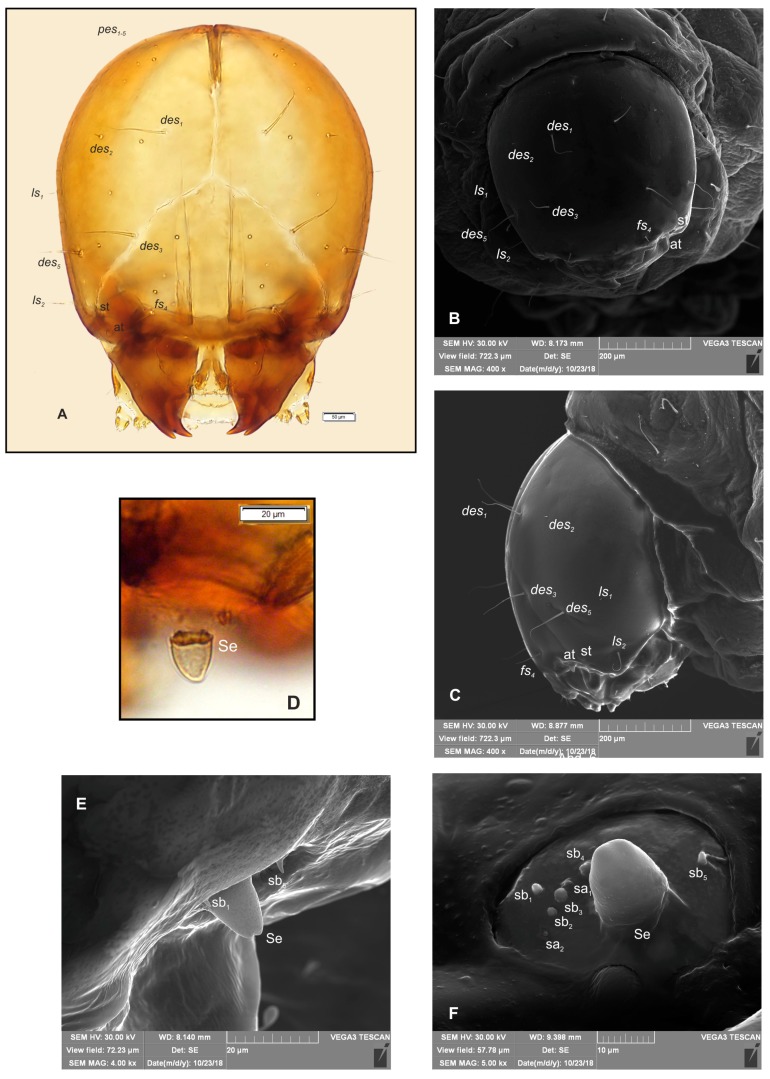
*Bagous claudicans* mature larva, head and antenna. (**A**)—head, frontal view; (**B**)—head, frontal view, SEM photography; (**C**)—head, lateral view, SEM photography; (**D**)—antenna, lateral view; (**E**)—antenna, lateral view, SEM photography; (**F**)—antenna, frontal view, SEM photography (at—antenna, sa—sensillum ampullaceum, sb—sensillum basiconicum, Se—sensorium, st—stemmata, setae: *des*—dorsal epicranial, *fs*—frontal, *ls*—lateral epicranial, *pes*—postepicranial).

*Head and antenna* (Figure 3A–F). Head light yellow, slightly narrowed bilaterally, frontal suture distinct, Y-shaped (Figure 3A). Setae on head trichiform, various in length. *Des_1_*, *des_3_* and *des_5_* elongated, equal length, *des_2_* very short, *des_4_* absent. *Des_1_* and *des_2_* placed in central part of epicranium, *des_3_* on frontal suture, and *des_5_* located anterio-laterally (Figure 3A–C). *Fs_4_* as long as *des_1_*, placed antero-laterally, close to epistoma. *Les_1_* very short, *les_2_* slightly shorter than *des_1_*. Post epicranial area has five very short *pes_1–5_* (Figure 3A). Frons with two pairs of pores placed medially; epicranium with two pairs of pores: one placed near to *des_1_*, next near to *des_5_*. Antenna located on end of frontal suture; antennal segment with Se elongated, located medially; basal membranous article with five sb and two sa (Figure 3D–F). 

**Figure 4:**
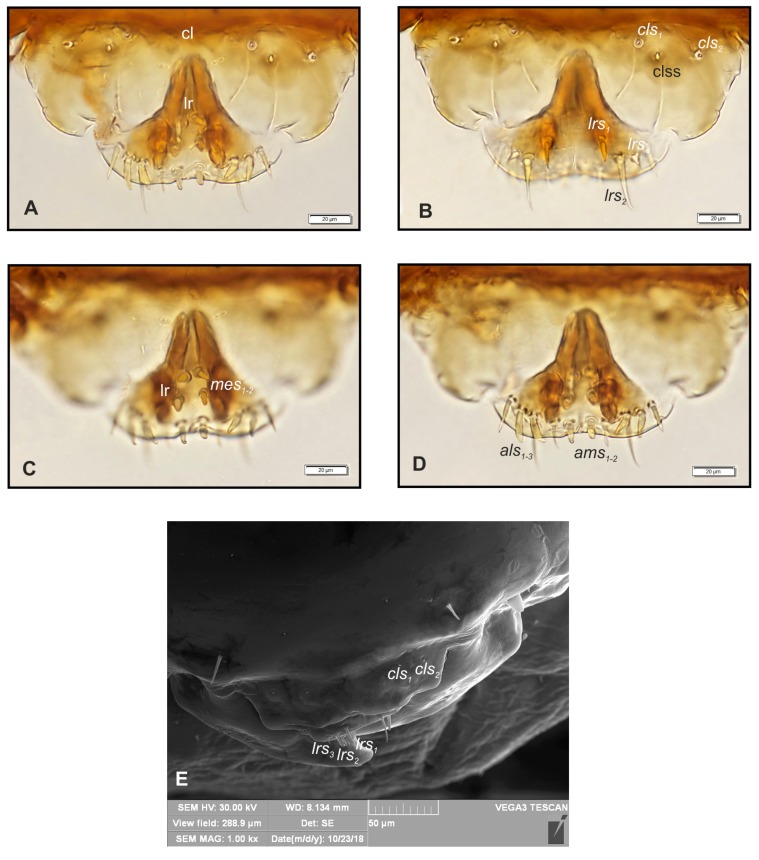
*Bagous claudicans* mature larva, clypeus, labrum and epipharynx. (**A**)—clypeus and labrum general view; (**B**)—clypeus and labrum (focused on dorsal surface); (**C**,**D**)—clypeus and labrum (focused on ventral surface); (**E**)—clypeus and labrum, SEM photography (clss—clypeal sensorium, lr—labral rods, setae: *als*—anterolateral, *ams*—anteromedial, *cls*—clypeal, *lrs*—labral, *mes*—median).

**Figure 5:**
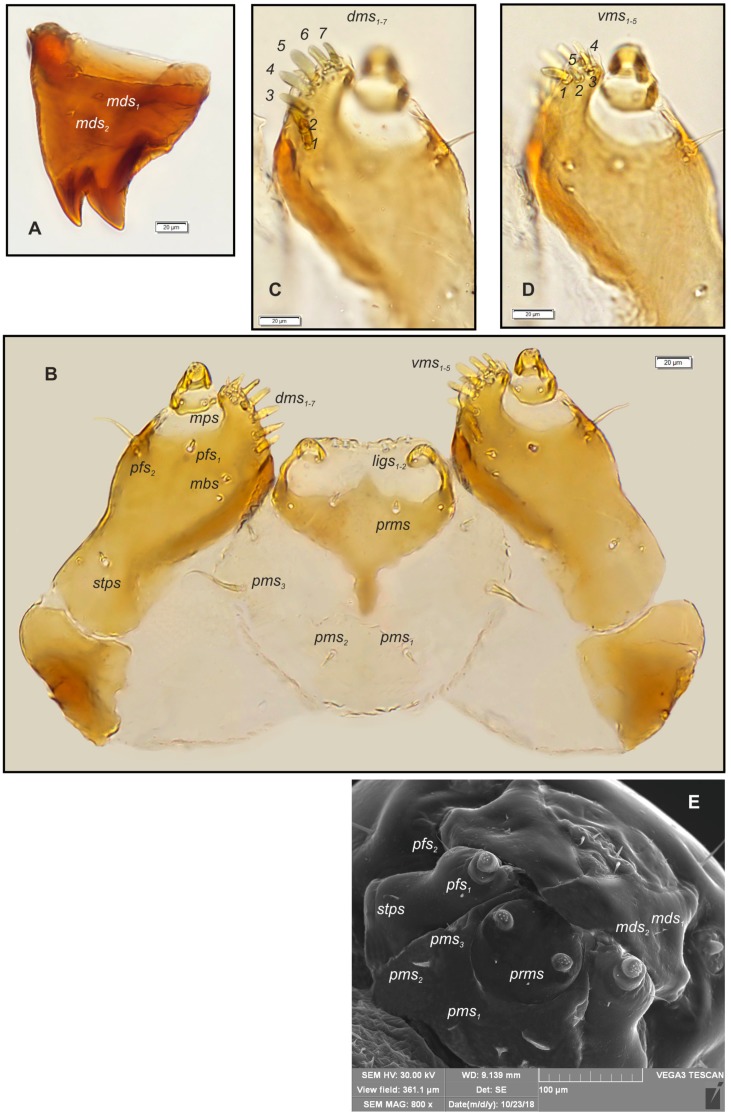
*Bagous claudicans* mature larva, mouthparts. (**A**)—right mandible; (**B**)—maxillolabial complex, ventral view; (**C**)—apical part of right maxilla, dorsal view; (**D**)—apical part of right maxilla, ventral view; (**E**)—mandibles and maxillolabial complex, SEM photography (setae: *dms*—dorsal malar, *ligs*—ligular, *mbs*—malar basiventral, *mds*—mandibular, *mps*—maxillary palp, *pfs*—palpiferal, *prms*—prelabial, *pms*—postlabial, *stps*—stipal, *vms*—ventral malar).

*Mouth parts* (Figure 4A–E, Figure 5A–E). Labrum approximately 3.0 times as wide as long, with three pairs of *lrs_1–3_* of various length; *lrms_1_* medium, *lrs_2_* very long, *lrs_3_* very short, all *lrs* placed medially (Figure 4B). Anterior margin of labrum slightly round emarginate. Clypeus 3.5 times as wide as long, with two short, triangular, and equally long *cls_1,2_* and one clss between them; all localized posterio-medially. Anterior margin of clypeus gently arcuated inwards. Epipharynx (Figure 4A, C–E) with three pairs of *als_1–3_* of various length (first and second finger-like, third the longest more hair-like); two pairs of short *ams_1,2_* (first short, finger-like; second medium length, hair-like) and two pairs of conical *mes_1,2_*. Lr elongated, slightly converging posteriorly (Figure 4A, C). Mandibles (Figure 5A) relatively broad, slightly truncate, bifid, teeth of unequal length, the cutting edge almost straight. Both *mds_1,2_* very short. Maxillary stipes (Figure 5B) have one short *stps* and two *pfs_1,2_* (first very short, second elongated); mala with one minute *mbs*, seven bacilliform or finger-like *dms_1–7_* of various length (Figure 5C); *vms_1–5_* various in length and shape (Figure 5D); *vms* always shorter than *dms*. Maxillary palpi with two palpomeres almost of equal length; basal with minute *mps* and a pore, apical with single pore. Apical part of distal palpomeres with a group of 5–6 elongated, conical, sensillae. Praelabium (Figure 5B,D) rounded, with a pair of short *prms* located medially. Ligula with two pairs of hairform, micro *ligs_1,2_* of equal length. Premental sclerite well visible, in a form of complete ring, with elongated proximal part. Labial palpi one-segmented; each palpomeres with a pore and some short, sensillae apically. Postlabium (Figure 5B,D) on ventral part densely covered by asperities and with three pairs of various in length *pslbs_1–3_* (first pair short, localized medially, second very long placed latero-medially, third very short, situated antero-laterally).

### 3.2. Description of Pupa of Bagous claudicans

**Figure 6:**
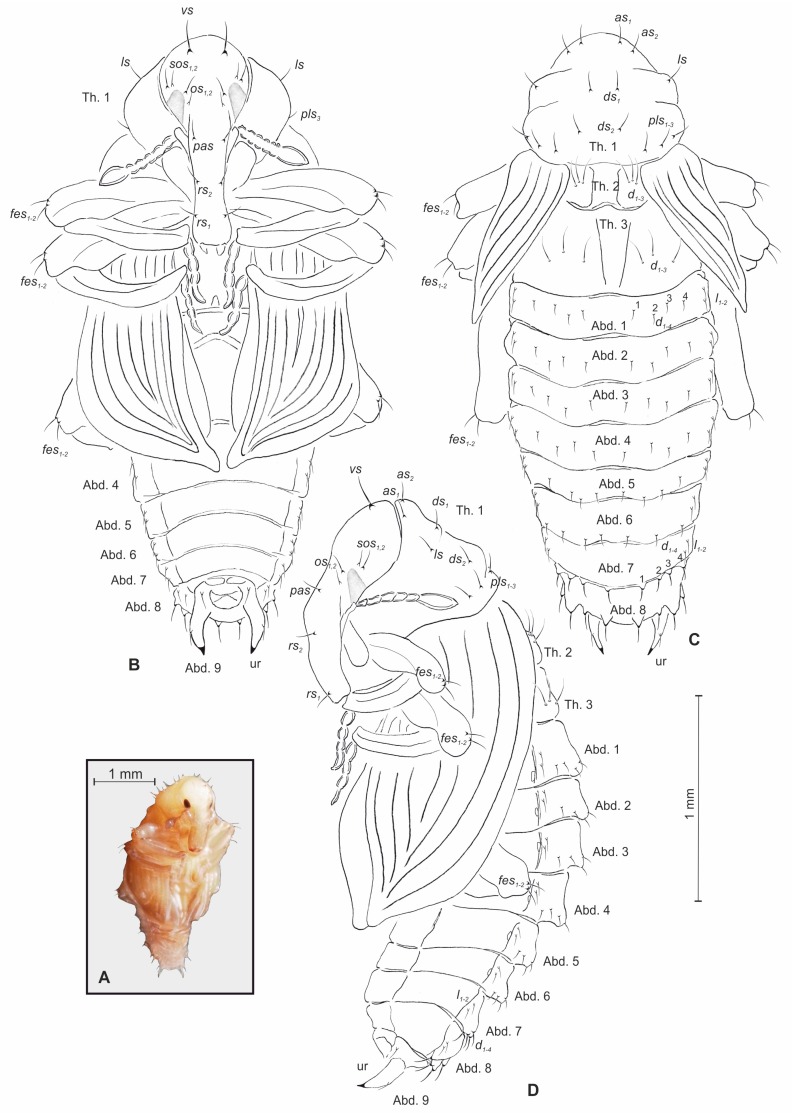
*Bagous claudicans* pupa. (**A**)—general habitus; (**B**)—ventral view; (**C**)—dorsal view; (**D**)—lateral view (Th. 1–3—pro–, meso– and metathorax, Abd. 1–9—abdominal segments 1–9, ur—urogomphus, setae: *as*—apical, *d*—dorsal, *ds*—discal, *fes*—femoral, *l*, *ls*—lateral, *os*—orbital, *pas*—postantennal, *pls*—posterolateral, *rs*—rostral, *sos*—superorbital, *vs*—vertical).

*General morphology* (Figure 6A–D). BL: 9.0 mm (♀), BW: 4.8 mm (♀), THW: 0.88 mm (♀). Body rather elongated, slender, white or yellowish. Cuticle smooth. Rostrum relatively long, approximately 5.0 times as long as wide, surpassing mesocoxae in repose. Antennae moderately long and slender. Pronotum almost as wide as long. Abd. 1–4 of almost equal length, segments 5–7 gradually diminished, 8th semicircular, 9th distinctly smaller than preceding ones. Urogomphia elongated and slender; each of them with sclerotized apex (Figure 6A–D). 

*Chaetotaxy*. Setae hair-like, of unequal length, yellow or light brown, on rostrum, head and pronotum based on small protuberances. Rostrum with two pairs of *rs_1,2_* (equal in length); head capsule bearing a pair of *vs*, two pairs of *sos_1,2_* (almost equal in length), two pairs of *os_1_,_2_* (various in length), and a pair of *pas* (Figure 6C, D). *Vs* distinctly bigger than remaining setae of head and rostrum. Pronotum on each side with two pairs of *as_1,2_*, a pair of *ls*, two pairs of *ds_1,2_*, and three pairs of *pls_1–3_*. All setae of pronotum almost equal in size (Figure 6C,D). Chaetotaxy of metathorax as on mesothorax, consisting of paired triplets *d_1–3_* (Figure 6C). Setae of meso- and metathorax very long, distinctly longer than setae of abdominal segments. Each femoral apex with a pair of *fes_1,2_* of almost equal length (Figure 6B–D). Each tergal part of Abd. 1–8 with four pairs of *d_1–4_* located medially, respective sternal parts of Abd. 1–8 with 2 pairs of *l_1,2_* located, close to the border with tergum. Dorsal and lateral setae of Abd. 1–8 very short, equal in length. Abd. 9 with two pairs of setae: first placed close to gonotheca, second on urogompia (Figure 6B–D). 

### 3.3. Genetic Results

**Figure 7:**
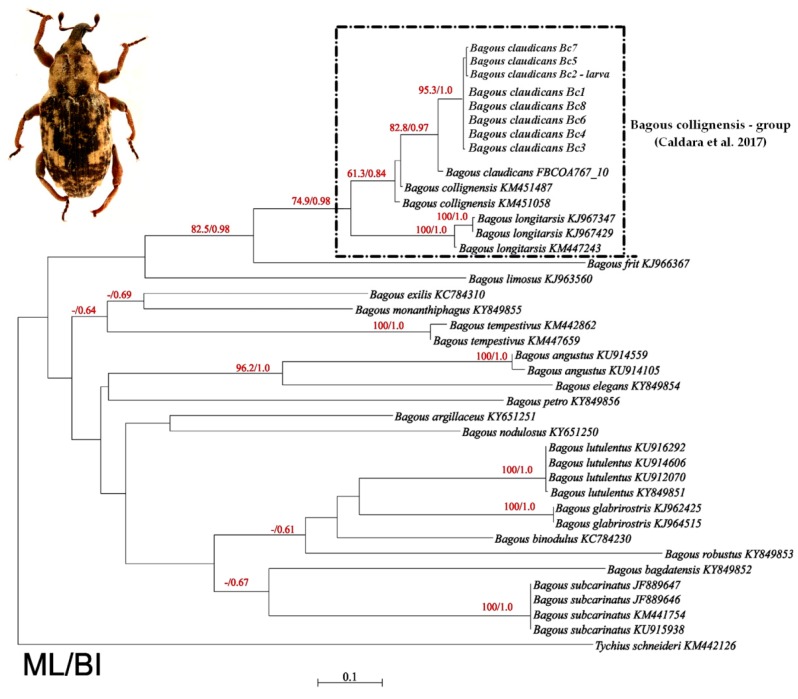
Phylogenetic tree of *Bagous* species inferred from maximum likelihood (ML) and Bayesian inference (BI) analyses of cytochrome oxidase I (*COI*) gene sequences. The first number on branch is bootstrap support from PhyML and the second is posterior probabilities from MrBayes. *Bagous claudicans* larva is marked..Codes after the species name are GenBank Accession Numbers. Scale bar unit: expected substitutions per site. Insert shows the dorsal habitus of an imago of *Bagous claudicans*.

Phylogenetic inferences were obtained for *COI* gene using Bayesian inference (BI) and Maximum likelihood (ML). The GTR+G+I model with gamma correction of 0.478 and invariable sites of 0.456 was selected by the AIC in jModelTest for the matrix. Heuristic searches resulted in one ML tree (−ln = −6064.16432). The Bayesian inference and maximum likelihood analyses resulted in similar trees, the only differences between them being the degree of statistical support for the recovered nodes (Figure 7). Nodal supports were generally poor across all backbone nodes. *Tychius schneideri* (Herbst) was used to root the topologies. The phylogram shows the existence of two clades. The first one formed by *Bagous limosus*, *B. frit*, *B. longitarsis*, *B. collignensis* and *B. claudicans.* The second had *B. claudicans* from Germany (based on data from BOLD Systems). The second clade consists of remaining *Bagous* species (Figure 7).

The *B. claudicans* forms in the first clade two phylogenetic lines, one with all newly investigated samples, both larva and imago from the Polish locality and the second with sample from Germany (based on data from BOLD Systems).

Based on the appropriate nucleotide substitution model, genetic distances between specimens ranged from 0% to 45%, while mean genetic distances between species ranged from 5% to 45% (Table 1).

## 4. Discussion

Dieckmann [59] placed *B. claudicans*, *B. collignensis*, *B. longitarsis*, and *B. rufimanus* in the middle European part of *B. collignensis* group. Also, valid classification [13] situates *B. claudicans* in *B. collignensis* group consisting of Palaearctic, North American and Indian species, including *B. bituberosus* LeConte, 1876, *B. nebulosus* LeConte, 1876, *B. pauxillus* Blatchley, 1916, *B. pusillus* LeConte, 1876, *B. confusus* O’Brien, 1995, *B. myriophylli* O’Brien, 1995, *B. diglyptus*, *B. longitarsis*, *B. lyali* Caldara and O’Brien, 1998, *B. riedeli* Caldara and O’Brien, 1998, *B. rotundicollis*, *B. rufimanus*, *B. tersus* Egorov and Gratshev, 1990 and *B. vivesi* González, 1967. Subsequently, based on characters of mature stages (e.g., structure of male genitalia and external morphology [21,59]), the current taxonomic position of *B. claudicans* seems to be adequate. On the other hand, its food preferences and host relations require further investigations.

Similarly, knowledge of morphology of immature stages of the *Bagous collignensis* group is still insufficient. From the above listed species’ preimaginal stages only three of them, *B. claudicans* (presented work), *B. collignensis* [18], and *B. rufimanus* [36], have been (more or less completely) described. Moreover, some previously published information appears to be inaccurate. De Meijere [60] described the larva of *B. claudicans* based on three exemplars collected in stem of *Equisetum limosum* L. (= *E. heleocharis*. Ehrh.). Subsequently, Scherf [18] published it as *B. collignensis* (treated *B. claudicans* as its synonym). But successive investigations never confirmed development of *B. claudicans* or/and *B. collignensis* on horsetails. According to the place and habitat, where the adults were collected, *B. claudicans* was classified as monophagous on *Equisetum limosum* [59], while *B. collignensis* as oligophagous on *Myriophyllum* L. [39,59,60,61]. The fact is, the only one of the *Bagous* developmental stages which were found on horsetails belonged to *B. lutulentus* [17]. This emphasizes that feeding of *Bagous* larvae caused very characteristic deformation on stems [20]. So, it seems to be very likely that material presented by De Meijere [60] and Scherf [18] concerns neither description of *B. claudicans* nor description of *B. collignensis*. Hence, most probably, larvae investigated by De Meijere [60] belonged in fact to *B. lutulentus*. Admittedly, there are some important differences between descriptions of larva of *B. claudicans* [59] (as *B. collignensis* [18]) and *B. lutulentus* Gosik (2009), e.g., stemmata absent; head narrow (1.39 as long as wide); *des_1_* and *des_2_* equal in length; Se conical [18] versus stemmata present; head semicircular (1.18 as long as wide); *des_1_* distinctly longer than *des_2_*; Se arrow-like [17]. However, it is to be noted that deformation of antenna, narrowing of head capsule, and consequently misinterpretation of chaetotaxy or (ostensible) disappearance of stemmata are belong to most typical results of invalid preparation of slides. On the other hand, habitus of the larval body showed by De Meijere [60] and Gosik [17] are very similar.

Based on existing descriptions, including Mantovani et al. [36], May [32], Cuppen and Heijerman [5], Staniec and Gosik [37], Gosik [17,34,35,36,38], Gosik and Wanat [34] and the presented work, the set of diagnostic characters for the larva of *Bagous* can be extended with the following items: (1) chaetotaxy of the body poorly developed; (2) *des_1_* occasionally absent (*B. aliciae*), *des_2_* reduced to absent, *des_3_* located on frontal suture, *des_4_* absent; (3) antenna with conical, more or less elongated *Se*; (4) epipharnyx with 1–2 pairs of *ams*, 2 pairs of *mes*, 3 pairs of *als*; (5) maxilla with 1 *stps*, 2 *pfs*, 3–7 *dms*, 1–4 *vms*; (6) stemmata present; (7) lr short thick; (8) ligula concave; (9) labial palp one segmented (10) lateral folds of Abd. 8 and 9 forming more or less visible protuberances; (11) spiracles of Abd. 8 placed dorsally.

Choosing the set of diagnostic characters for the immature stages of *Bagous collignensis* group at this stage (valid descriptions of only two species) is not desirable. Nevertheless, several characteristics typical only for *B. claudicans* and *B. rufimanus* can be listed in larval stage: antenna with 8 sensillae vs. 6 or less on other *Bagous; pms_2_* at least twice longer than *pms_1_* and *pms_3_* vs. *pms_2_* as long as others or only slightly longer; *mbs* present vs. *mbs* absent (except *B. brevis* and *B. binodulus*). And in pupal stage: *os_1_* and *os_2_* various in length vs. equal in length (if present) on other *Bagous* (except *B. elegans*); 3 pairs of *pls* vs. always 2 pairs of *pls*; 1 pair of *ls* vs. 2 or 3 pairs of *ls* (except *B. frivaldszkyi*).

On the other hand larvae of *B. claudicans* and *B. rufimanus* are different each other in larval stage in: shape of head (rounded on *B. claudicans* vs. narrowed bilaterally on *B. rufimanus*), postdorsum of abdominal segments 1–7 with 4 setae on *B. claudicans* vs. 2 setae on *B. rufimanus*; epipharynx with 2 pairs of *ams* on *B. claudicans* vs. a single pair on *B. rufimanus*. And in pupal stage by: 2 pairs of *rs* on *B. claudicans* vs. a single pair on *B. rufimanus*; lack of *sls* on *B. claudicans* vs. 2 pairs of *sls* on *B. rufimanus*.

The pupa of *B. claudicans* doesn’t show any new characters different from those compiled by Gosik and Wanat [34] for *Bagous*.

Interestingly, the larvae, pupa and imagines of *B. claudicans* have been collected not from *Equisetum*, but instead from the stems and roots of the decorative variety of *Sedum maximum*, growing in the center of a city (Katowice). *Sedum* Linnaeus, 1753 plantings grew under unfavorable conditions: on a thin, dry substrate (insulated with a geomembrane), in an exposed area, and under strong insolation. This site was more akin to xerothermic environments than to humid habitats settled by most species from the genus. Thus, the treatment of *B. claudicans* as a monophagous of *Equisetum* is certainly not based on full knowledge of its biology and requires more detailed study. Detailed observations of this species are, however, very difficult due to its rarity and similarity to closely related *B.collignensis*.

Results from phylogenetic analysis confirm that investigated larva and imagines belonged to *Bagous claudicans* species, which was additionally supported by comparing them with the known sequence from BOLD Systems (Figure 7).

Moreover, the performed phylogenetic analysis indicates that *Bagous longitarsis* is a sister group to *B. collignensis* and *B. claudicans*, which confirms the previous results obtained by Caldara et al. [13].

Despite the value of the mean genetic distance between *B. claudicans* and *B. collignensis* (5%, Table 1), which may indicate that the investigated species can belong to the same species presented in this study, morphological and ecological data suggest the existence of two separate biological species. Also *B. longitarsis* are genetically close to *B. collignensis* (8%) and *B. claudicans* (12%), all these species are grouped in close related *collignensis*-group distinguished by Caldara [13] (Table 1).

Therefore, the hypothesis of Scherf [18] that *B. claudicans* is a synonym of *B. collignensis* must be further investigated, especially using a large number of specimens from different localities as well as additional markers, as the genetic distance within *B. claudicans* can range to 3% on specimens from different localities (Germany vs. Poland). Moreover, further phylogenetic studies of all the known *Bagous* species are required in order to understand the relationships within the *Bagoini* tribe.

## 5. Conclusions

Morphological characteristic of immatures of *Bagous claudicans* (in both, larval and pupal stages) are typical for the genus *Bagous*, especially for *B. colligensis* group. At the same time, some original features make possible distinguishing of *B. claudicans* from other known *Bagous* species. Additionally, host plant and ecological preferences of *B. claudicans*, meticulously analyzed during presented study, emphasize taxonomical distance between *B. claudicans* and *B. collignensis* (which was previously questioned). Furthermore, DNA barcoding confirms not only larval identification but also its usefulness in specimen identification of larval stages Moreover, it seems, that advanced study on morphology of developmental stages and ecology of *Bagous* are required in order to clarification some of systematic ambiguities and efficient protection of the genus.

## Figures and Tables

**Table 1:** Mean genetic distances (%) between included *Bagous* species. Genetic distances based on a GTR + I + G model of DNA evolution. Species from the *Bagous claudicans*-group in bold, red font.

**Species Name**	***B. rgillaceus***	***B. angustus***	***B. bagdatensis***	***B. binodulus***	***B. claudicans***	***B. collignensis***	***B. elegans***	***B. exilis***	***B. frit***	***B. glabrirostris***	***B. limosus***	***B. longitarsis***	***B. lutelentus***	***B. monanthiphagus***	***B. nodulosus***	***B. petro***	***B. robustus***	***B. subcarinatus***
***B. argillaceus***																		
***B. angustus***	31																	
***B. bagdatensis***	36	36																
***B. binodulus***	30	38	28															
***B. claudicans***	29	39	36	37														
***B. collignensi***	27	36	36	38	**5**													
***B. elegans***	29	24	37	30	38	34												
***B. exilis***	24	35	32	28	28	24	33											
***B. frit***	30	41	45	38	22	23	40	30										
***B. glabrirostris***	27	40	32	16	45	40	34	30	39									
***B. limosus***	33	38	32	35	28	24	31	23	32	42								
***B. longitarsis***	27	38	38	38	**12**	**8**	31	26	25	43	27							
***B. lutelentus***	32	38	30	18	42	39	33	36	44	21	33	40						
***B. monanthiphagus***	24	30	32	24	28	28	30	21	28	26	31	30	27					
***B. nodulosus***	27	30	27	31	34	35	32	24	38	35	32	34	32	25				
***B. petro***	27	30	38	31	34	37	26	36	31	34	36	31	32	26	29			
***B. robustus***	32	38	37	23	42	42	36	43	40	25	38	40	27	29	37	31		
***B. subcarinatus***	25	31	24	25	31	30	36	35	39	27	38	32	30	29	28	37	34	
***B. tempestivus***	28	31	27	30	29	27	34	27	35	34	31	30	41	26	31	32	39	31
